# Autopsy findings in victims of gunshot injuries: wound ballistic considerations based on test shots to simulants and composite models

**DOI:** 10.1007/s00414-025-03543-w

**Published:** 2025-06-27

**Authors:** Markus Große Perdekamp, Stefan Pollak

**Affiliations:** Institute of Forensic Medicine, Faculty of Medicine, University of Freiburg, Albertstraße 9, 79104 Freiburg, Germany

**Keywords:** Experimental wound ballistics, Gunshot injury, Simulants, Ordnance gelatine, Glycerin soap, Artificial bone, Composite model, High-speed videography

## Abstract

The focus of this review is on the experimental reproduction of gunshot injuries as observed in real autopsy cases. At the centre of considerations are the morphological features and diagnostic conclusions derived from the wound appearance. With this in mind, the technical possibilities to simulate the tissue damage are described on the basis of the pertinent literature and our own studies. In particular, the following issues are addressed: (1) the morphology of bullet entrance wounds depending on the shooting distance (contact, close range and distant shots); (2) the findings along the bullet path (with special regard to different soft tissues and the fracture patterns in bones); (3) the use of biological and synthetic simulants as target material; (4) imaging techniques and evaluation methods in one-component surrogates and composite models; (5) injuries from uncommon weapons and shooting devices.

## Introduction

The morphological appearance of a gunshot injury reflects the dynamic interaction between the bullet and the tissues penetrated along its path through the body. The transfer of energy causes deformation, destruction and displacement of the affected structures resulting in local damage with consecutive bleeding. Due to the great number of influencing factors, both on the part of the projectile and the target, no wound pattern exactly resembles another. Nevertheless, it is reasonable to expect that the individual injury allows conclusions to be drawn about the main characteristics of the causative gunshot. It is, therefore, no surprise that a bullet’s behaviour and its effects on a specific target were already studied under standardized conditions a long time ago. As targets a wide range of objects including animal bodies, isolated organs, homogeneous simulants and composite models were used.

The following sections exclusively focus on the morphological features of gunshot wounds. Any impact on physiological functions such as a victim’s ability to act, the ‘stopping power’ and the lethality remains unconsidered in this context. Instead, the findings at the bullet entrance site, along the intracorporal wound channel and at the point of exit are looked at by comparing real cases with test shots to surrogates. So the starting point for explanatory approaches is the analysis of particular findings in real gunshot injuries and their imitation in test sets simulating the wounding mechanism.

## Gunshot entrance wounds

### Distant shots

In distant shots, entrance wounds are characterized by three main features: the central bullet hole in the skin, the abrasion collar and the ring of dirt (provided that no intermediate target was hit).

#### Bullet holes

Already in the early twentieth century, most comprehensive textbooks of forensic medicine (e.g. [1]) emphasized that the size of the permanent bullet entrance hole does not permit conclusions as to the calibre of the gun. In experimental studies conducted by Sellier [2] it was demonstrated that the skin is radially accelerated and displaced centrifugally when passed by the bullet head. As a result, the wound diameter is temporarily larger than the calibre of the projectile. The radial displacement is, however, partly reversible in elastic tissues causing a skin hole that is mostly smaller than the bullet’s cross section. With regard to synthetic skin, the consecutive phases of the penetration process have been recorded by Thali and coworkers [3].

In a recent study by Geisenberger et al. [4], porcine skin from the anterior and posterior trunk was shot at using the same ammunition. On the back with its comparatively thick dermis, the skin defects were considerably smaller than those of the ventral trunk where the corium is less thick. The significant difference can be explained by the fact that the elastic properties of the skin are mainly determined by the connective tissue due to its richness of fibres.

Former investigations [5] disclosed that the palmar and plantar regions are body areas where gunshot holes are disproportionally small. The discrepancy between the size of the bullet head and the entrance defect has been attributed to the particular stratification and elasticity of hairless skin. Surprisingly small entry holes have also been recorded in nail plates consisting of keratin [6]. The same applies to highly elastic material of non-human origin such as rubber, whereas inelastic targets such as modelling clay reveal entrance defects exceeding the bullet’s calibre [4].

To investigate the correlation between the design of the bullet and the size of the respective skin hole, experimental shots were fired to porcine specimens [7]. Shots with wadcutter projectiles produced larger defect areas than truncated cone and round nose bullets.

Jussila et al. [8] evaluated a selection of synthetic and natural materials as potential skin simulants by analysing their mechanical and ballistic properties. A semi-finished cowhide of 0.9–1.1 mm thickness was found to be particularly suitable. Recently, Kerkhoff et al. [9] proposed a combined cowhide/gelatine soft tissue simulant for ballistic studies and forensic shooting incident reconstructions. Roebuck 1518 synthetic chamois has also been validated as a suitable skin simulant [10]. Based on extensive studies, Fischer et al. [11] recommended dental silicone Shore hardness 70 as an appropriate skin simulant for wound ballistic examination. Several studies into skin simulants have provided criticism, as the reported energy density at perforation is very inconsistent [12].

Re-entry shots were simulated on the basis of cylindrical skin-gelatine models [13]. When there was a distance between the first and second segment, the re-entry skin wound resembled the primary entrance hole in size and appearance. In test shots with a contact between the two segments, the tissue defect at the re-entry site was surrounded by irregularly defined areas of epidermal loss, and in some cases the skin holes were accompanied by short radial tears. As Haag and Jason [14] demonstrated, common pistol bullets exiting from a primary target are mostly destabilized. Nevertheless, the entrance wounds in secondary gunshot victims may appear as typical first-strike lesions, if the bullets retain their nose-forward flight.

**Fig. 1:**
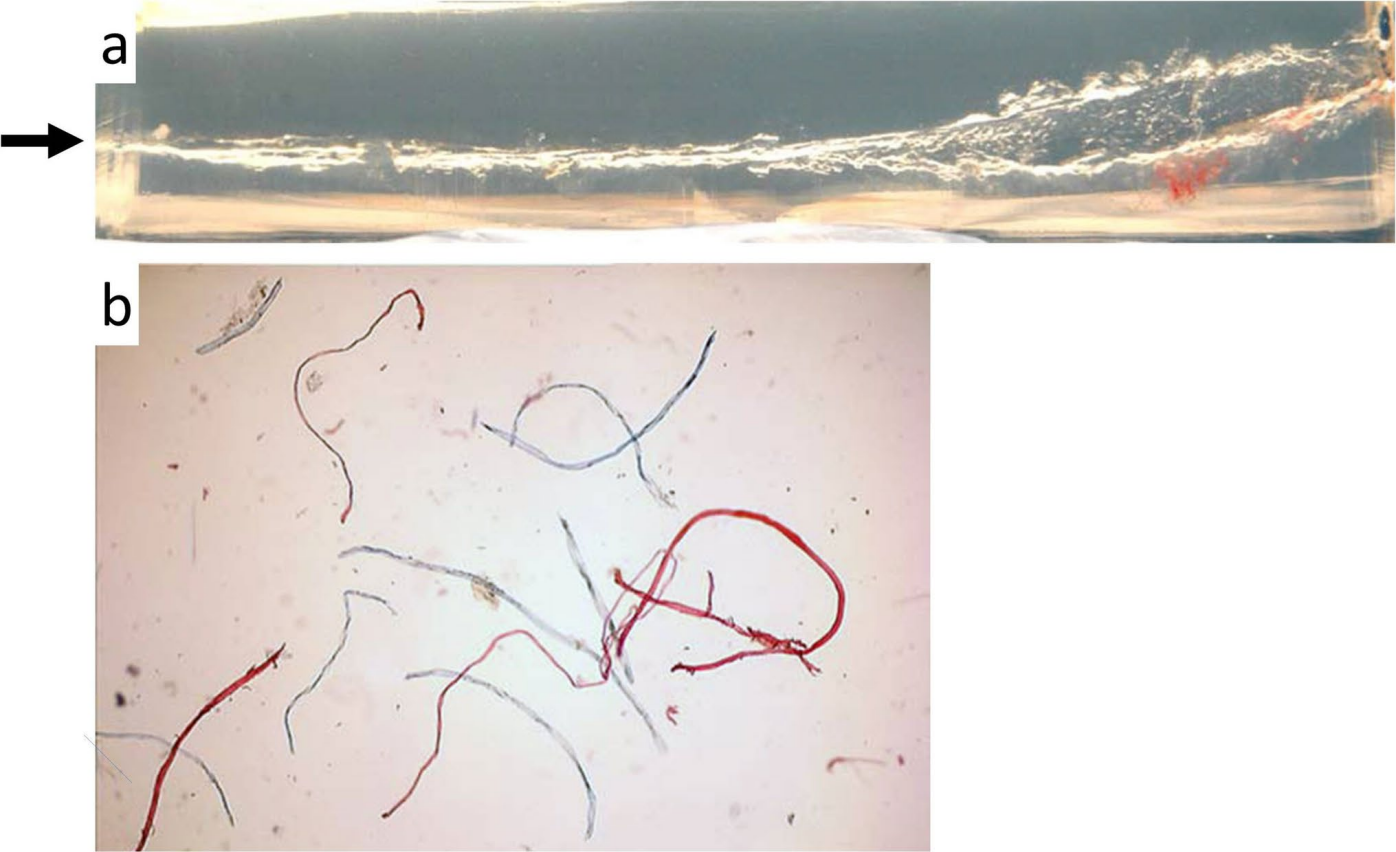
(cf. [16]) **a** Transmitted light print of a 25-cm-long bullet track in a gelatine block. The direction of the shot is indicated by an arrow. The entrance site (on the left) was covered with a blue cotton textile and the exit (on the right) with a red one. Near the bullet exit, red fibres transferred in a retrograde manner are visible to the unaided eye. **b** Microscopic demonstration of displaced jeans fibres from the entrance region (blue) and the exit region (red) in the bullet track 15 cm away from the entry (× 100 magnification)

In order to answer the question what happens to the tissue lost at the entrance wound, experimental studies were performed on composite models consisting of dyed pig skin and gelatine blocks [15]. For the test shots, cartridges with different bullet types were used. In all skin-gelatine preparations, coloured skin particles from the entrance site were demonstrated along the whole bullet track. It can thus be assumed that the tissue lost at the entry is fragmented and mostly displaced in the direction of the shot. The same applies for textile fibres from the anterograde transport, whereas fibres from the exit region may be transferred back into the bullet path [16] (Fig. 1).

#### Abrasion collar

The epidermis-free margin, which typically encircles the bullet entrance hole, is mostly called abrasion collar though this term may lead to false associations. The name derives from a time when it was wrongly assumed that the bullet indents and thereby abrades the skin when penetrating [17].

As early as the sixties of the last century, Sellier [2, 18] presented the results of his test shots to skin preparations. With the help of high-speed photography he could prove that the loss of epidermis around the entrance hole was not caused by any skin indention with consecutive friction. As a matter of fact, superficial particles from the wound edges are thrown back against the direction of fire. Since then, the findings published by Sellier have been reproduced in further studies (e.g. [6, 13, 15, 19]).

In the plantar and palmar regions characterized by a thick horny layer, a circumferential loss of epidermis is usually missing. Instead, the stratum corneum bordering on the bullet entrance hole is lifted from its base and torn radially [5].

Systematic investigations of inclined shots to skin preparations revealed, as expected, an eccentric broadening of the abrasion collar on the side from which the shot was fired [19]. Within this zone being prone to drying-up, a tongue-shaped area of preserved epidermis could be shown by histological examination.

Findings resembling an abrasion collar are not specific to bullet entrance wounds in humans. They may also occur when non-biological targets with a layered surface such as painted metal sheets are shot at [20]: The superficial coating is lost around the central hole resulting in a ring-shaped area exposing the underlying substrate.

The working group of Thali et al. [3] described the temporal development of entrance wounds by firing test shots to a ‘skin-skull-brain model’ and recording the bullet’s penetration with a high-speed camera. To simulate the human skin, a silicon cap containing synthetic fibres was used [21]. Upon contact with the head of the projectile, the skin surrogate moved laterally and outwardly, in a cone shape, opposite to the direction of shot. The authors argued that the abrasion ring is caused by temporary overstretching of the skin adjacent to the penetration point. Backspatter of surface particles was not to be expected as the synthetic skin of the model was single-layered in contrast to human and porcine skin which is composed of epidermis and corium.

The morphological features of re-entry shots were examined using skin-gelatine composite models [13] simulating the consecutive passage of a bullet through two parts of the body. If the exit and re-entry site were in contact, the corresponding skin wounds resembled each other as both had central tissue defects surrounded by abrasion areas.

#### Bullet wipe (‘ring of dirt’)

The term refers to the ring-shaped blackish discolouration immediately adjacent to bullet holes and mostly overlying parts of the abrasion collar, provided that the (unclothed) skin was the primary target. As already stated in early textbooks (e.g. [1]), soot carried by the projectile is wiped off at the site of impact. Apart from combustion residues of the propellant, primer elements (such as lead, barium and antimony) as well as any weapon lubricant are further constituents of the dirt deposits. Based on experimental studies, Elbel [22] could demonstrate that the bullet wipe is more pronounced in shots from freshly oiled weapons compared with shots from lubricant-free barrels. If the primer contains a lead compound, a positive colour reaction with sodium rhodizonate is to be expected.

In test shots to the glabrous skin of palms and soles, it turned out that gunshot residues may be deposited under the detached epidermis constituting an ‘internal ring of dirt’, regardless of the firing range [23].

Test shots were fired to blood-soaked garments to clarify whether this condition possibly prevents the formation of a bullet wipe around the entrance hole [24]. In shots to fabrics oversaturated with blood, a bullet wipe was lacking, whereas lead-containing particles were spotted in the periphery.

Arrow entrance holes from crossbows may be surrounded by a blackish ring resembling the bullet wipe in gunshots. As experimental shots to composite models showed, the material deposited circularly on the wound margins is consistent with that of the arrows’ tips and carbon shafts [25].

### Contact and close-range shots

The following features are regarded as typical of contact entrance wounds: (1) the presence of a muzzle imprint mark, (2) a powder cavity underneath the skin and (3) the facultative occurrence of skin tears radiating from the entrance hole (especially in regions having a bony support).

**Fig. 2:**
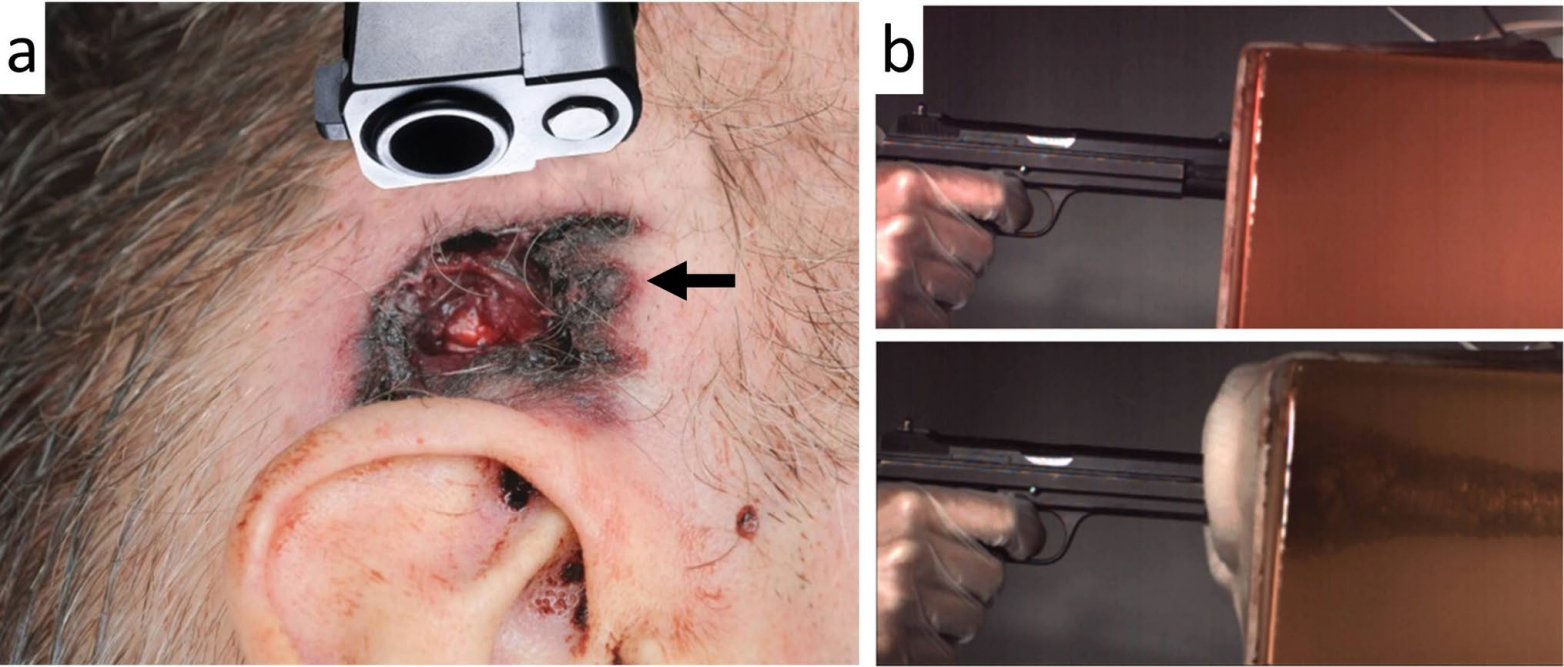
(cf. [33]) **a** Contact shot to the head (entrance site above the right auricle). The weapon used was a pistol Smith & Wesson M&P 45. The imprint mark of the recoil spring guide (arrow) is particularly pronounced. **b** Experimental contact shot to a gelatine block coated with pig skin and a polyurethane plate underneath (to simulate a bony support). The picture below shows the maximum expansion of the bloated skin after 5.7 ms

The *muzzle imprint* (barrel marking) was described by Werkgartner already in the 1920s [26–28]. According to his observations, the muzzle mark is a pressure abrasion, whose shape often depicts the contours of the barrel end and the surrounding construction parts of the weapon. The underlying mechanism was clarified by experimental studies of Hausbrandt [29] and Elbel [30], who by means of a high-speed camera was able to prove that the bullet entrance site balloons backwards against the muzzle due to the expansion of the inrushing combustion gases (Fig. 2). Apart from the common abrasion type, the patterned muzzle imprint may also be constituted of intradermal blood extravasations [31].

Thali et al. [32] documented the dynamic development of the muzzle imprint by using a non-biological skin-skull-brain model: High-speed photography confirmed that the skin surrogate bulges backwards against the muzzle. Pircher et al. [33] reported on the findings in experimental contact shots to composite models. According to their results, the imprint mark of semi-automatic pistols may differ from the shape of the muzzle plane insofar as the retractable parts are either not depicted or only to a minor degree. If this is the case, the muzzle imprint preferentially mirrors the front sides of the stationary parts such as the barrel end, the recoil guide and the gun housing.

The ‘*pocket*’ (‘soot cavity’) under the skin contains soot and gunpowder particles. It is rightly regarded as a significant indicator of a contact shot [17]. Already in 1890, Paltauf [34] recognized that in contact and some close-range shots both blood and muscle tissue may assume a bright red colour due to the formation of carboxyhaemoglobin and carboxymyoglobin.

In a study on porcine soft tissue [35], gunshot residues deposited in the depth of the bullet entrance site could be detected by means of 3-dimensional CT. Schyma et al. [36] fired contact shots to containers filled with gelatine and coated with a silicone layer. High-speed videography revealed that the powder cavity rises in about 1.5 to 2 ms whereas its collapse takes 2.5 to 3 ms. The size of the silicone dome increased with decreasing barrel length. In rifles and shotguns, the effect of a shortened barrel is similar to that in handguns: Due to the higher gas pressure at the muzzle, the expansion in the first section of the bullet track is increased. This phenomenon was reproduced by test shots to soap blocks with consecutive CT scanning [37].

**Fig. 3:**
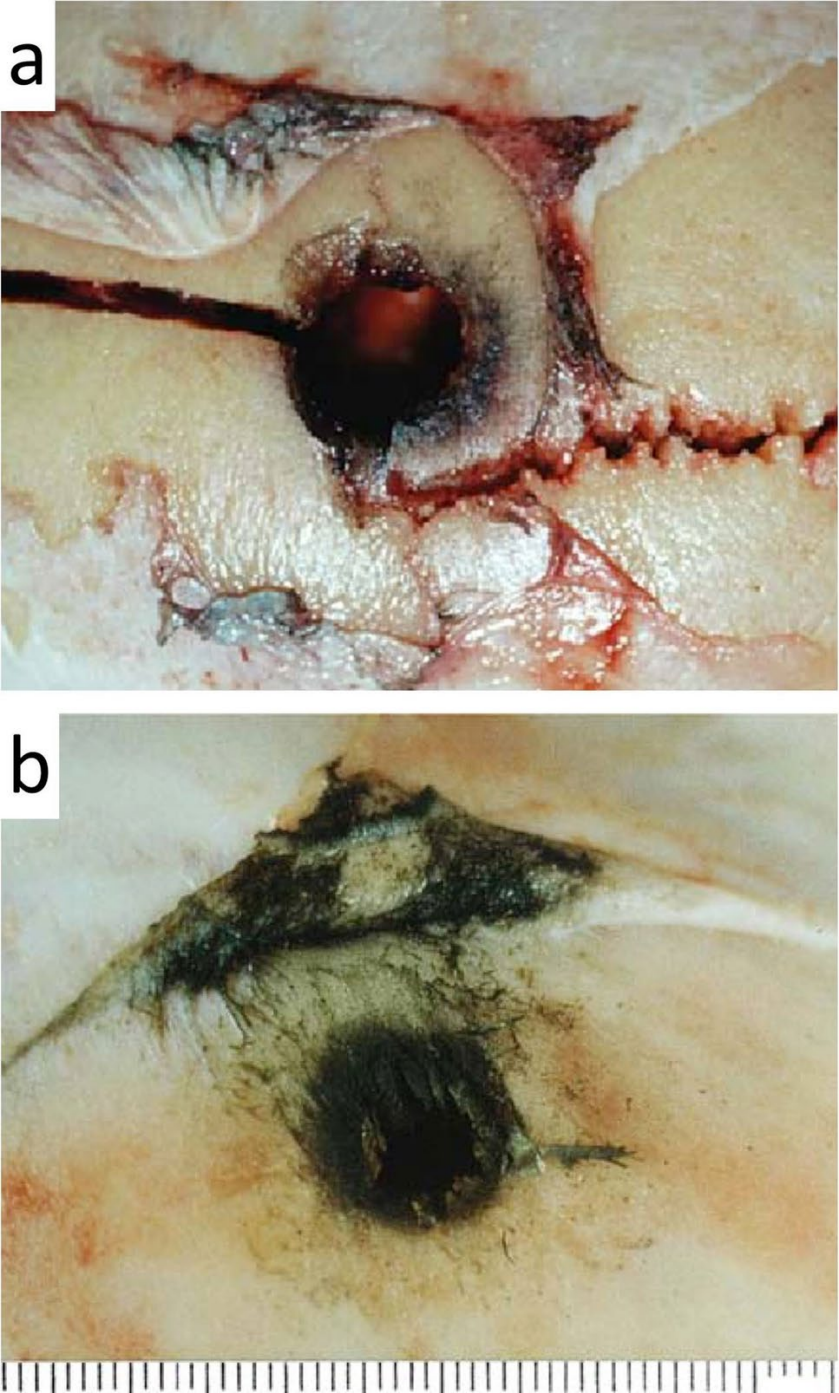
(cf. [38]) **a** Findings in the depth of a contact entrance wound (parietal region, pistol cal. 7.65 mm). Soot staining of the outer table around the bullet hole. The periosteum is detached, with its blackened underside turned outward. **b** Experimental contact shot to the forehead of a slaughtered calf. After removal of the epicranial soft tissues, a circular zone of soot staining is visible around the bullet hole. The periosteum is detached; its blackened underside has been turned upward during dissection

In contact shots to the cerebral cranium, the periosteum around the bullet hole in bone may be detached and reflected with soot staining on the underside [38]. Analogous findings were obtained in experimental shots to the frontal bone of a slaughtered calf (Fig. 3).

**Fig. 4:**
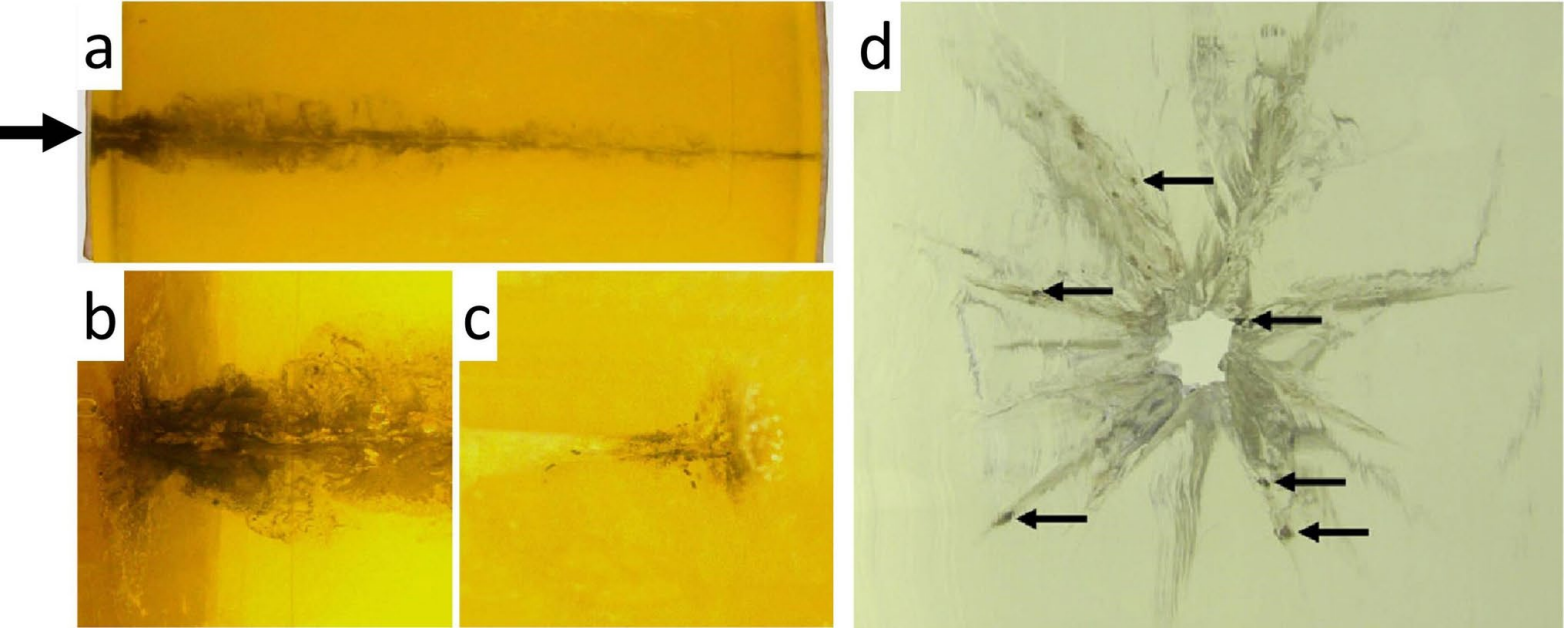
(cf. [39]) Contact shot to a composite model consisting of a gelatine block coated with pig skin at the entrance and exit sites. The direction of the shot is indicated by an arrow. The weapon used was a pistol Heckler & Koch cal. 9 × 19 mm. **a** Transmitted light print of the 25-cm-long bullet track. **b** The initial section shows intense blackening. **c** Final section containing powder particles. **d** Axial view of the permanent bullet track with radiating cracks highlighted by soot. The arrows point to gunpowder particles

Autopsies on victims who were injured by a contact shot revealed that the presence of soot, powder particles and carboxyhaemoglobin may not be restricted to the initial parts of the wound track. In experimental shots to composite models simulating soft tissue targets, discharge residues including primer elements could be found along the whole bullet path [39] (Fig. 4). The temporal propagation of the sooty material was visualized by high-speed videography.

**Fig. 5:**
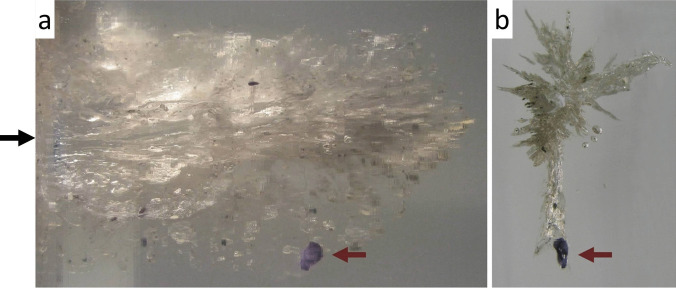
(cf. [42]) Effect of the muzzle gases from a blank cartridge in a contact shot to a composite model (gelatine block coated with dyed pig skin on the front side). The black arrow indicates the direction of fire. The weapon used was a pistol Walther P 22. **a** Lateral view of the gelatine block, transmitted light print. **b** Axial view of a gelatine layer with star-shaped slits containing soot and a skin particle (red arrow)

It is a well-known fact that contact shots from blank cartridge handguns may cause penetrating and even fatal shots [40, 41]. The wound morphology resembles the findings in contact shots with live ammunition regarding the muzzle imprint mark, the powder cavity and bright red tissue discolouration from the formation of carboxyhaemoglobin as well as the facultative presence of stellate tears at the entrance site. According to test shots fired at composite models of pig skin and gelatine, the penetration depth in the simulant amounted to several centimetres [42]. Similar wound cavity depths were measured under the experimental conditions chosen by Euteneuer et al. [43]. The skin particles from the entrance, together with the gunshot residues, are dispersed in the affected gelatine, especially the radial slits (Fig. 5).

**Fig. 6:**
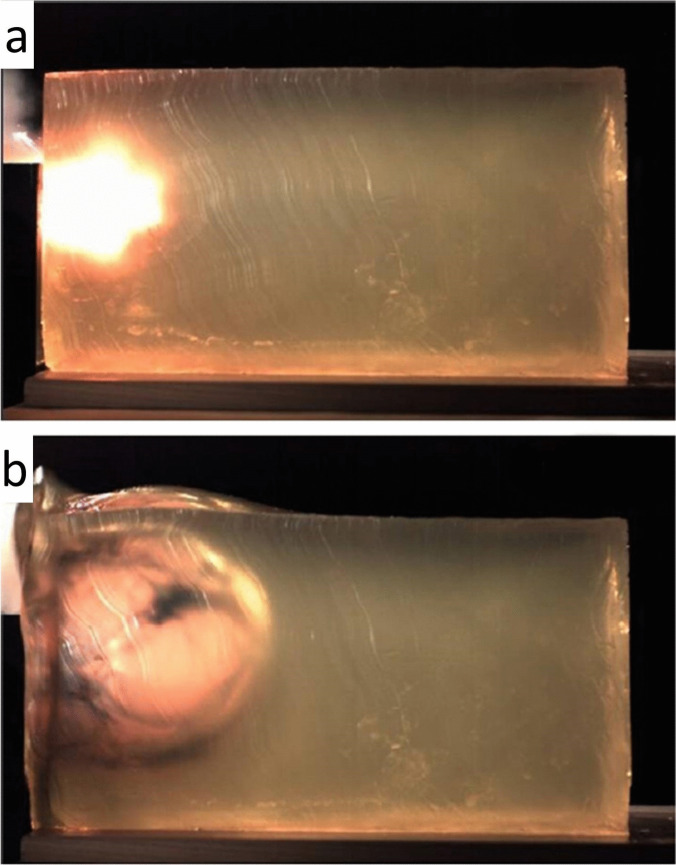
(cf. [44]) High-speed video documentation of a contact shot to a gelatine block. The weapon used was a muzzle-loading pistol discharging only black powder (5 g) and no bullet. Size of the temporary cavity in the initial phase **(a)** and at the moment of maximum extent **(b)**

Apart from conventional handguns, contact shots with muzzle-loading black powder pistols may cause fatal injuries even if no bullet but only the propellant is discharged. In the latter case, the wounding effects result from the high energy density of the gas jet. With the help of test shots to gelatine blocks it was possible to reproduce the shape and extent of the tissue damage in a particular incident [44] (Fig. 6).

Schyma [45] performed experiments to clarify whether the muzzle gases invading the tissues in contact shots have an additional wounding potential. The test shots were fired to head models (acryl spheres filled with gelatine and covered with a layer of silicone). The findings proved a more extensive destruction of the simulant in contact shots compared to close-range and distant shots.

*Stellate tears* radiating from the bullet entrance hole are a characteristic, but not obligatory feature of contact shots, especially in body regions having a bony support. The wound shape depends, among other factors such as the charge strength, on the surface structure of the underlying bone [46].

In contact shots to a non-biological skin-skull-brain model, Thali et al. [32] could reproduce radial tears of the artificial skin due to its retrograde bulging by the inrushing combustion gases.

The shape and pattern of gunshot residue (GSR) depositions in near-contact and contact gunshot wounds was studied by means of test shots to a skin and soft tissue model [47]. As might be expected, the powder soot halo enlarged with increasing muzzle-target distance, whereas its density declined. In angled shots, the area of intense powder soot blackening was eccentric, elliptical in shape and pointing toward the muzzle.

In distant shots to fabrics saturated with fluid blood, a great number of rhodizonate positive particles may be scattered around the bullet entrance thus wrongly simulating GSR from a close-range shot [24].

## Wound morphology within the body

### General aspects

The extent of destruction along consecutive sections of a bullet path is mainly influenced by the physical properties of the projectile and its interaction with the different tissues passed through. Therefore, in terminal ballistics most surrogates simulate either soft tissues or bones or combinations of both. In composite models, the main body is often covered with animal skin (mostly taken from slaughtered pigs) or with a synthetic skin surrogate. If ballistic gelatine is used as soft tissue simulant, parenchymatous organs of animal origin such as liver, spleen, kidney or lung may be embedded to provide a realistic model composed of different body components. This also applies to combinations with hard substances like (synthetic) bones or teeth.

### Soft tissue simulants

**Fig. 7:**
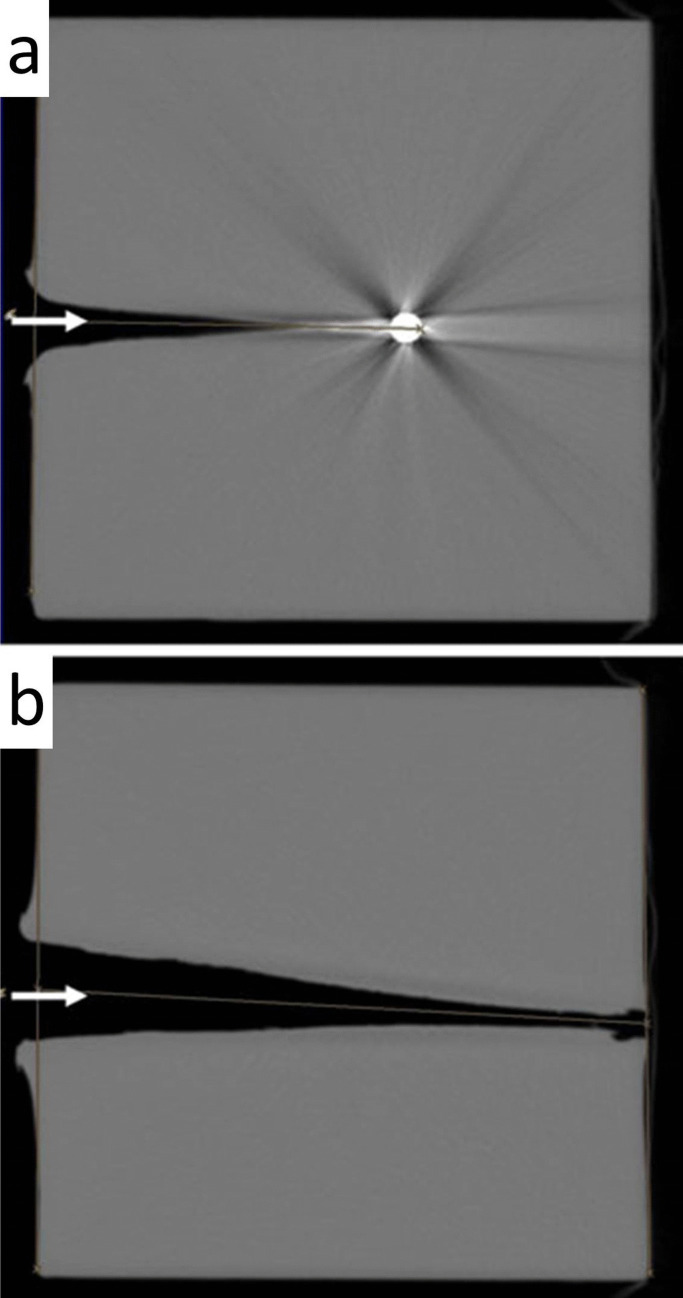
(cf. [50]) Contact shots into ballistic glycerin soap using different charges of black powder (1.5 and 2 g, respectively). The entrance sites are marked by arrows. The cavitations caused by the spherical lead balls are visualized by computed tomography. Note the conical shape of the cavitations. In the upper image **(a)**, the bullet got stuck after a distance of 15 cm (1.5-g charge). In the lower image **(b)**, the projectile travelled through the whole thickness of the block (25 cm)

In principle, a distinction has to be made between surrogates with elastic properties (gelatine being the main example) and non-elastic simulants with plastic behaviour such as glycerin soaps, clay or plasticine. Both groups of target media are characterized by the fact that their density is similar to human organs and other soft tissues regarding the energy transfer of penetrating bullets. In elastic materials, the lateral acceleration resulting in a temporary cavity (TC) is mostly reversible in contrast to non-elastic simulants in which the maximum extent of the TC is ‘frozen’ in the absence of restoring forces. Due to the translucency of gelatine, in this surrogate the spatial–temporal development and size of the TC may be documented by means of high-speed video-recording. If a soap block is fired at, the TC can be made visible and measured either by cutting open the target material or by computed tomography (CT), which allows a non-destructive examination [48, 49]. In contrast to conventional (elongated) bullets, the cavitations caused by spherical projectiles have a conical shape [50] (Fig. 7).

The configuration and dimensions of the permanent cavity have been recorded by analyzing CT scans of gelatine blocks shot at with an assault rifle section by section [51]. Based on the CT images, the wound channel’s profile was found to be narrow elliptical corresponding to the devitalized tissue zone subjected to surgical debridement in casualties.

The dynamic behaviour, the penetration depth and the deformation of a bullet are very similar in gelatine and glycerin soap [52]. From a physical point of view, there is no reason to prefer one simulant. Therefore, the choice of simulant should depend on the respective requirements of the particular study.

Nowadays, ballistic gelatine is rightly considered the current gold standard for human soft tissue surrogate material [53]. It has been widely used in terminal ballistics research since the 1980s [54–57]. The manifold aspects of preparation and standardization [52, 53, 58–61] are dealt with in specialist scientific publications being outside the scope of this review, which primarily focuses on the interpretation and experimental reconstruction of the morphological findings in real gunshot wounds.

Apart from high-speed videography and CT imaging, the cracks radiating from the permanent wound channel are used as an indicator of the energy transferred by the bullet. For assessing consecutive slices of gelatine cut at right angles to the trajectory, different types of measurement have been proposed: the total crack length (TCL) method [62], Fackler’s wound profile [54] (taking into account the two largest cracks) and the polygon technique (by linking the ends of the cracks) [63].

The working group of Bolliger [64] suggested to use non-destructive computed tomography (CT) as a means of determining the gunshot energy transfer. The authors applied the total crack length method along the projectile’s course in blocks of ballistic gelatine. Based on the CT data, the lengths of all fissures were measured in 1-cm steps permitting the creation of an energy transfer profile typical for the given bullet design.

In order to enhance the visibility of the radial cracks and to facilitate their length determination, Schyma [63] fired test shots to gelatine blocks marked with acryl paint pads on the front. As the paint soaked into the simulant, the colour contrast accentuated the fissures and made it easier to measure the lengths of the radial tears.

A further study by Schyma [65] investigated the correlation of the TC (recorded by high-speed video [HSV]) and the cracks in gelatine slices by firing test shots with common handguns and different bullet types. According to the results presented, the TCs caused by full metal jacketed (FMJ) projectiles were tubular (indicating a continuous deceleration of the missiles), whereas in shots with hollow-point (HP) bullets the TC configuration was pear-like. The maximum of TC stretching observed in HSV did not coincide with the maximum gelatine destruction as demonstrated with the crack length method.

**Fig. 8:**
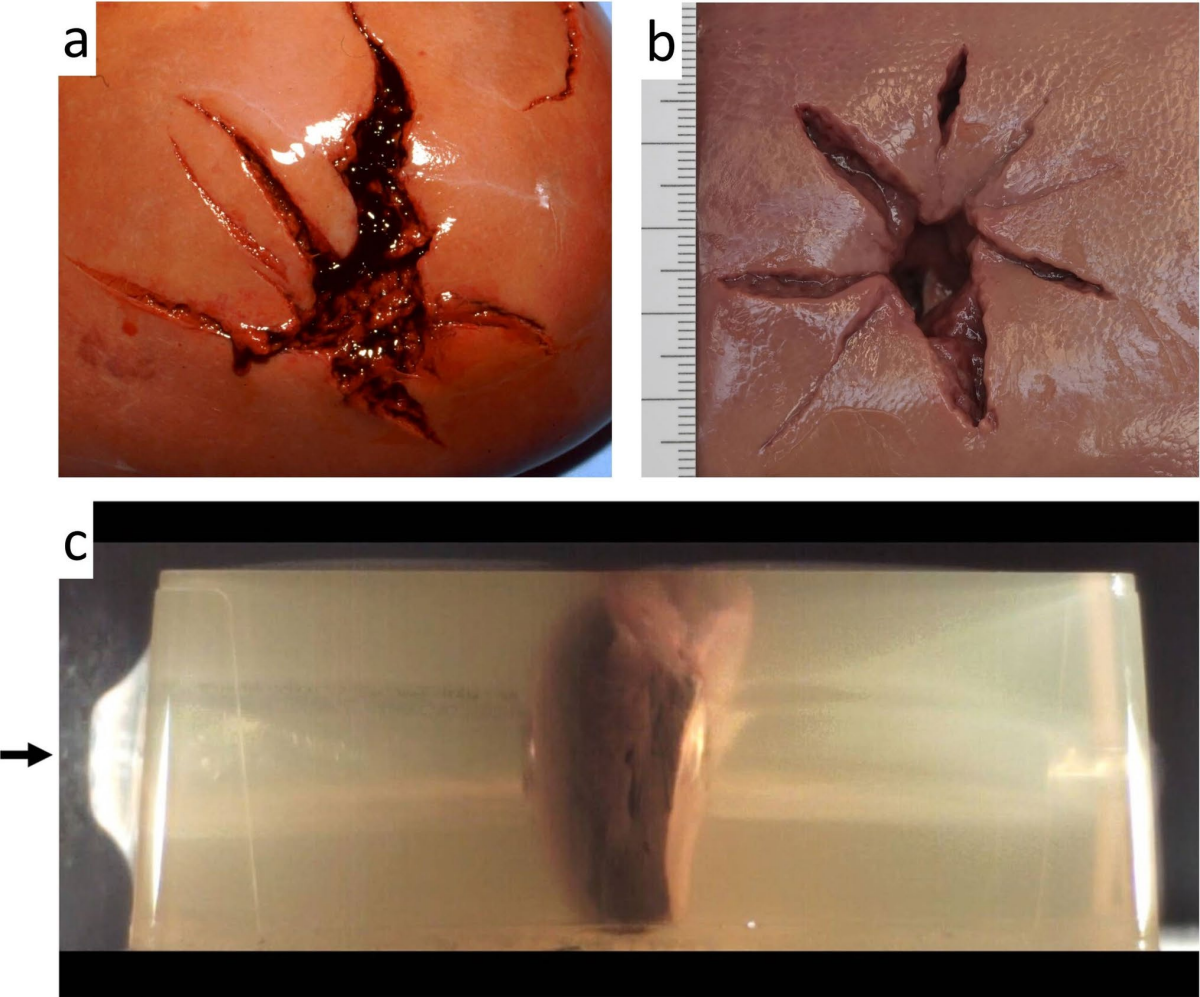
(cf. [67]) **a** Bullet entrance wound of the liver in a fatally injured gunshot victim (pistol ammunition 9 mm Luger). **b** Porcine liver showing a stellate entrance wound inflicted post-mortem. **c** High-speed video recording displaying the temporary cavity along the bullet's path through a composite model containing porcine liver embedded in gelatine

It has been stressed that the dimensions of the TC do not necessarily correspond with the extent of tissue damage along the bullet path. Organ-specific textures have a major influence on the interaction with the bullet and the resultant injury pattern [66]. Shots to composite models demonstrated that gunshot wounds in liver, spleen and kidneys had a star-shaped appearance, whereas wound channels in pulmonary tissue were tubular and lacked additional cracks [67] (Fig. 8). The differing behaviour can be explained by the unequal density of the affected tissues. Stellate wounds in liver and spleen have been reproduced experimentally by shooting at isolated cadaver organs [68]. Corresponding observations have been reported by Fackler et al. [69] for the Russian AK-74 assault rifle.

Anatomical structures having a high elasticity such as non-sclerotic aortic walls may also show stellate lacerations. In real cases and in test shots to isolated preparations, the bullet entrance and exit holes were disproportionally small and accompanied by radiating tears [70].

Maiden et al. [71] conducted energy loss experiments on porcine organs using steel 4.5 mm BBs. The results obtained with heart and lung were similar to those with FBT specification gelatine, whereas spleen resembled the NATO specification. Mabbot et al. [72] examined the damage caused by 0.223 Remington rounds both in simulated porcine thoraces and in gelatine blocks. The authors concluded that the damage produced in thoraces was smaller than in gelatine.

Humphrey et al. [73] compared the energy loss of steel spheres in soft tissue simulants with that in porcine specimens (lung, liver, kidney, heart). The study results suggested that the existing simulants do not fully represent the physical properties of real organs.

Experimental pistol shots were fired at soft tissue simulants to demonstrate any deflection of the trajectory. Deflection was small to virtually absent with wound channel lengths of 5 and 10 cm, but increased considerably with lengths of 15, 20 and 25 cm [74].

Bolliger et al. [75] produced a non-biological lung model as a possible alternative to organs from body donors or slaughtered animals. The lung substitutes were made of frothed ordnance gelatine which was cooled to stable foam blocks. The radiological and physical densities as well as the wound profile characteristics were comparable to real lungs.

Dental alginate and agar impression materials were examined for their suitability as brain simulants [76]. When comparing the two surrogates tested, agar behaved more like brain in terms of damage and energy absorption. The working group of Zhang [77] used spherical head models with Sylgard® (a silicone elastomer) as a brain simulant. Lazerjan et al. [78] investigated the mechanical properties of different brain simulants to analyze the mechanisms of backspatter in gunshots to the head.

Oehmichen et al. [79] evaluated the zones of cellular and axonal destruction around the permanent wound track in cases of low-velocity gunshots to the head. Microscopic examinations demonstrated axonal injury even at sites remote from the permanent bullet track. It is a well-known fact that the brain surface may show contusions far away from the actual wound channel, obviously due to the interaction with the encasing cranial vault. Gunshot-induced contusions are mostly located on the bases of the frontal and temporal lobes [80]. Similar to indirect skull fractures, cerebral contusions are attributed to enhanced pressure effects within the confined space of the neurocranium. According to an evaluation of case studies [81], immediate incapacitation is to be expected in gunshots associated with cortical contusions.

### Simulation of human hard matter

Real gunshot injuries of human bodies mostly affect not only soft tissues but also hard matter, namely bones or – less frequently – teeth. The comparatively high density of those materials influences both the projectile and the target substances in many ways: The bullet velocity is slowed down to a greater extent, the energy transfer is increased, the maximum expansion of the TC takes place at an earlier point in time, the penetration depth is shortened, bones are subjected to direct and/or indirect fragmentation. A bullet that hits bone may be deflected and/or tumble already in the initial section of the wound channel. The projectile itself is often deformed or even disintegrated.

According to Kneubuehl [52], a bone simulant should fulfil the following requirements: (1) similar deceleration to that in real bone, (2) similar threshold velocity for penetration and (3) similar fracture behaviour. Nowadays, most artificial bones are made of polyurethane, e.g. Synbone® (Synthetic Bone For Surgical Education). Anatomical models are often composed of a cancellous inner core and a harder shell imitating the exterior cortex, the latter being possibly covered with a thin layer of latex as a surrogate for the periosteum. Artificial bones may be designed either as simple geometric forms (e.g. plates, hollow cylinders and spheres) or as models with lifelike shapes. If hollow bones are used as targets, the marrow can be simulated by gelatine [82]; otherwise the passing bullet would not generate the hydraulic pressure necessary to cause the typical fracture pattern. Similarly, hollow spheres representing the skull are often filled with gelatine to simulate the brain (e.g. [21, 83]).

A bone surrogate may be used as single target material (to study exclusively its interaction with the bullet) or as part of a compound model (together with a soft tissue simulant). Already in the more distant past, particular attention was paid to gunshot wounds of flat bones, especially those of the cranial vault [17]. Di Maio [84] fired test shots to isolated human bones to find out the minimum velocity required by a bullet to effect penetration of flat bone from the skull cap (thickness 4–6 mm). According to his data, perforation of the bone was the rule at about 90 m/s. In Synbone® surrogates, the mean velocity to produce fractures from direct loading was 146 ± 3 m/s [85].

It has been known for a long time that bullet holes in the skullcap are characterized by cone-shaped widening on the exit side. The entrance is usually round to oval and sharp-edged, whereas the opposite surface on the exit side is bevelled thus indicating the direction of the shot. Occasionally, bullets may cause minor bevelling also on the entrance side [86, 87].

A study by Kuhl and Janssen [88] demonstrated that the entrance holes produced by jacketed bullets are mostly smaller than, or at most identical with, the diameter of the projectile. In shots with lead bullets, the bone defects were slightly larger than the calibre. Similar results were obtained in test shots to extracranial bones of predominantly spongy structure such as the ala ossis ilii and the corpus sterni [89].

In a study published by Taylor and Kranioti [83], the radius of the entrance hole in polyurethane proxies was positively correlated with the calibre dimension. As it turned out in another investigation by Taylor et al. [90], bovine scapulae can be appropriate for ballistic simulations of flat bone injuries as the wound morphology is similar to that in humans. Experimental shots from military rifles to synthetic skull proxies (Synbone® spheres) filled with ballistic gelatine revealed that the entry wound morphology closely resembled real forensic cases [91].

Tangential shots to the cranial vault typically result in gutter-like bone wounds [84]. If the projectile strikes the skull at a shallow angle, a so-called keyhole lesion is to be expected [84, 92, 93]. When looked at from the outside, the bone defect is composed of (1) a rounded section with a sharp edge and inner table bevelling (pointing towards the weapon) and (2) a triangular area of external bevelling on the opposite end. An experimental study on cranial bones [93] revealed that keyhole-shaped fractures may also occur in shots perpendicular to the skull.

Thali et al. [94] fired grazing shots with FMJ and lead bullets to artificial bone simulating the cranial vault. The different kinds of interaction between the two bullet types and the bone surrogate were recorded with a high-speed camera. The FMJ projectile did not suffer an apparent deformation. By contrast, the lead bullet was split in two parts. In orthogonal shots, the entrance holes were cratered on the inner surface just like the ones in real cases.

Riva et al. [95] compared the findings obtained in test shots to synthetic head models with real gunshot wounds. According to the authors’ investigations, the perforation and penetration capacities were comparable. On the other hand, differences were observed with regard to the bullet deformation. A further study of Riva et al. [96] dealt with the practical application of synthetic head models for the comparison with CT and autopsy findings in victims of real gunshot fatalities. In order to reproduce the wound characteristics, two types of head models (‘open shape’ and ‘spherical’) have been assembled using leather (as skin surrogate), polyurethane (as bone simulant) and gelatine (as substitute for brain tissue). With both the ‘open shape’ and the ‘spherical’ model it was possible to imitate a bullet’s intracranial ricochet.

Gunshots to the head are often accompanied by linear skull fractures radiating from the bullet entrance and/or exit hole. In addition, the bone defects may be encircled by concentric fracture lines. Indirect fractures away from the permanent wound channel are mainly present in particularly thin parts of the cranium such as the orbital roofs and the temporal squamae. The indirect fractures are attributed to the shot-induced pressure increase within the confined space of the skull. All kinds of bullet holes and the fractures that may be associated with them have been reproduced in experimental settings using head models [83].

In an experimental study conducted by Mahoney et al. [97], Synbone® spheres filled with ballistic gelatine were shot at with 7.62 × 39 mm FMJ ammunition (mean impact velocity 654 m/s). The overall fracture patterns were judged as being too comminuted when compared with actual military head injuries.

Another study undertaken by Mahoney et al. in 2017 [98] dealt with anatomically correct skull-brain models. Test shots with 7.62 × 39 mm FMJ ammunition produced fracture patterns similar to those found in real cases. Anatomically correct head models have also been used to generate backspatter in contact and intermediate-range shots [99].

Apart from gunshots to the cranial vault and other flat bones, experimental injuries have been inflicted to human and animal long bones as well as synthetic surrogates imitating their original structure [82, 100, 101]. The periosteum may be simulated by a layer of latex and the surrounding soft tissues by embedding the bone in gelatine. Bone marrow is also substituted by ballistic gelatine. The bullet’s energy transfer results in bone fragmentation and is often accompanied by additional deformation of the projectile.

**Fig. 9:**
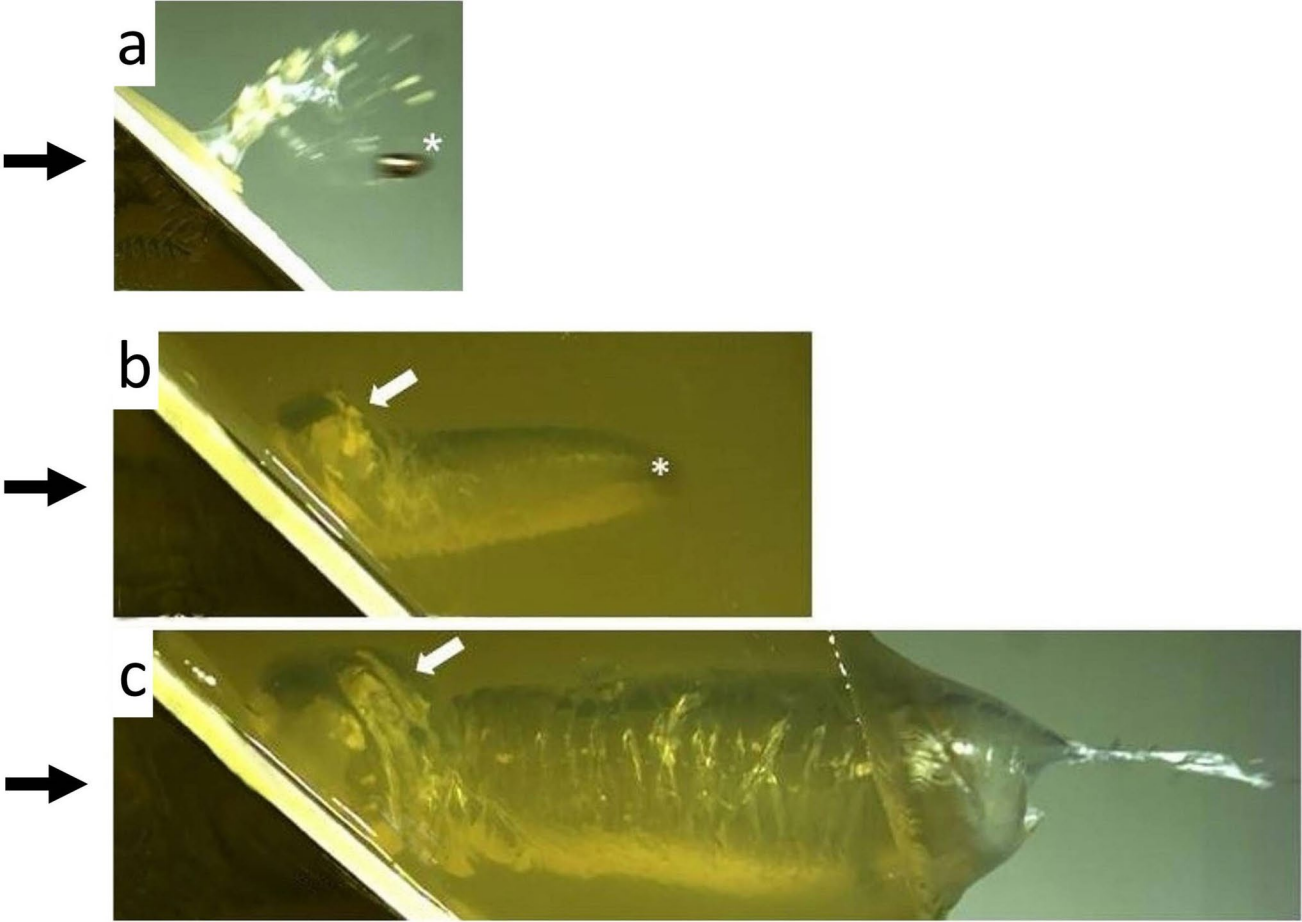
(cf. [102] **a** Short time imaging of an angled shot fired with a pistol cartridge cal. 9 mm Luger to a synthetic bone plate. Most bone particles move at right angles to the plate, whereas the projectile (*) is followed only by some smaller splinters. The black arrows indicate the direction of fire. **b, c** Angled shot through a bone plate embedded in gelatine. The high-speed images show the temporal development of the cavitation. The asterisk in Fig. 9b marks the position of the bullet. In Fig. 9c, the projectile has already left the composite model. The white arrows point to an eccentric bulge of the temporary cavity caused by the osseous fragments moving at right angles from the perforated synthetic bone sheet into the gelatine

In shots to porcine and synthetic bones, the fragments travel forward and opposite to the direction of fire. With both targets, the dimensions of the TC correspond to each other. Bone fragments are located within the extent of the TC and the permanent wound track [82, 102] (Fig. 9).

Schwab et al. [100, 101] fired test shots to shafts of human femurs and described the fracture patterns depending on the anatomical location and the angle of impact. The bullet entrance sites displayed roundish holes with radiating fractures, marginal chipping and wing flake defects. The mean horizontal entry hole diameter exceeded the projectile’s calibre.

Pullen et al. [103, 104] compared experimental shots to porcine ribs and Synbone® plates using two kinds of ammunition. The Synbone plates turned out to be a good proxy for flat bone with no significant difference between ribs and 5 mm plates.

### Composite models

In the recent past, a large number of simple and complex models have been created in order to simulate the interaction between the bullet and a combination of different tissue surrogates simulating the specific anatomical conditions of the injured body part [105–108]. Most experimental settings try to imitate an individual shooting incident. Accordingly, the test design varies from case to case. The examples presented in the following are, therefore, by no means exhaustive.

Considering the wound channel’s profile, the size of a bullet exit wound has to be discussed with regard to its spatial relation with the TC. To study this topic, test shots using 5.56 × 45 mm cartridges were fired to composite models consisting of gelatine and porcine tissue covered with skin at the exit site [109]. The position of the TC in relation to the exit plane was decisive for the size of the skin wound: If it was located within the cavity, the wound dimensions were much larger compared to exits in front or behind the TC.

**Fig. 10:**
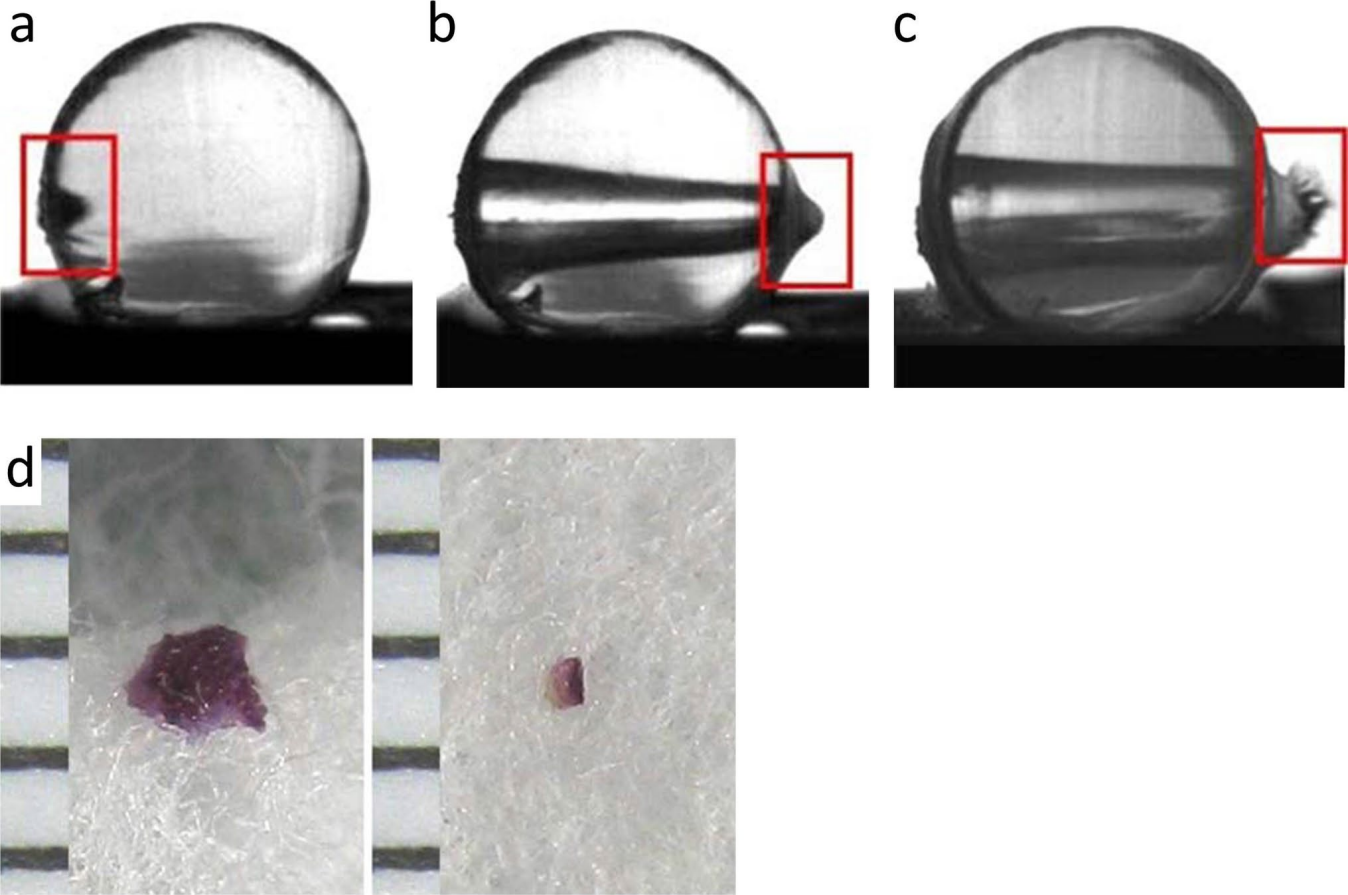
(cf. [13]) Distant shot to a cylindrical gelatine block wrapped in pig skin. The weapon used was a pistol Heckler & Koch USP cal. 9 × 19 mm. The high-speed image recording shows the bullet penetrating into the composite model **(a)** and just before emerging from the protruding exit site **(b)**. In Fig. 10c, the skin of the exit area has burst outward; the temporary cavity extends from the entrance to the exit. **d** Stereomicroscopical views of a backspatter particle originating from the skin of the entrance site (left half of the image) and a horny scale deriving from the exit and displaced in the direction of fire

By recording the exit wound formation with a high-speed camera, the outward bulging and local overstretching of the skin can be made visible (Fig. 10). The slit-like or stellate appearance is plausibly explained by the circular hyperextension of the tissue followed by radiant tearing.

Bullet fragmentation observed in autopsy cases has successfully been reconstructed with the aid of composite models. For example, it has been shown that FMJ bullets fired from an assault rifle may disintegrate after contact with teeth [110]. Another ballistic model simulated a bullet fragmentation due to an intermediate target, in particular an interposed finger [111]. Gascho et al. [112] reproduced the distribution pattern of metallic fragments from a disintegrated bullet that was prevented from exiting by hard material.

In some ways, a protective helmet worn on the head may be regarded as an intermediate target modifying the injury pattern. The effect of helmet materials on bullet behaviour and cranio-cerebral injury was examined using various head models. Mahoney et al. [113] fired 7.62 × 39 mm FMJ bullets at simplified head models supplemented by an additional layer simulating a combat helmet. This resulted in a greater variability of the TC dimensions.

As the topic of protective helmets and other kinds of body armour is related rather to military medicine than to the field of forensic medicine and criminalistics, the current review only mentions some relevant references [114–120].

As it was rightly stressed by Maiden [121], experimental models simulating soft tissues and bones have shown that gunshot injuries can be reconstructed with considerable accuracy. Individual synthetic head models have been compared with CT and autopsy findings in real cases [95, 96]. By combining various surrogates it is possible to simulate human tissues in their spatial anatomical context – a key requirement for reproducing a specific bullet-body interaction.

Apart from gunshot wounds in a narrow sense, injuries from *shooting devices* such as livestock stunners and nail guns can be made the object of model-based research. In slaughterer’s guns, a cylindrical steel bolt is driven into the head by firing a blank cartridge. After having penetrated the braincase, the bolt is pulled back into the barrel (usually by rubber bushings and/or a withdrawal spring). In some powder-activated captive-bolt stunners, the soot is drained off through smoke conduits opening in the muzzle plane [122–125].In angled shots, the shape and location of any smoke depositions indicate the inclination of the barrel. Similarly, a unilaterally bevelled edge of the entrance wound points to the direction of the fire [124].

**Fig. 11:**
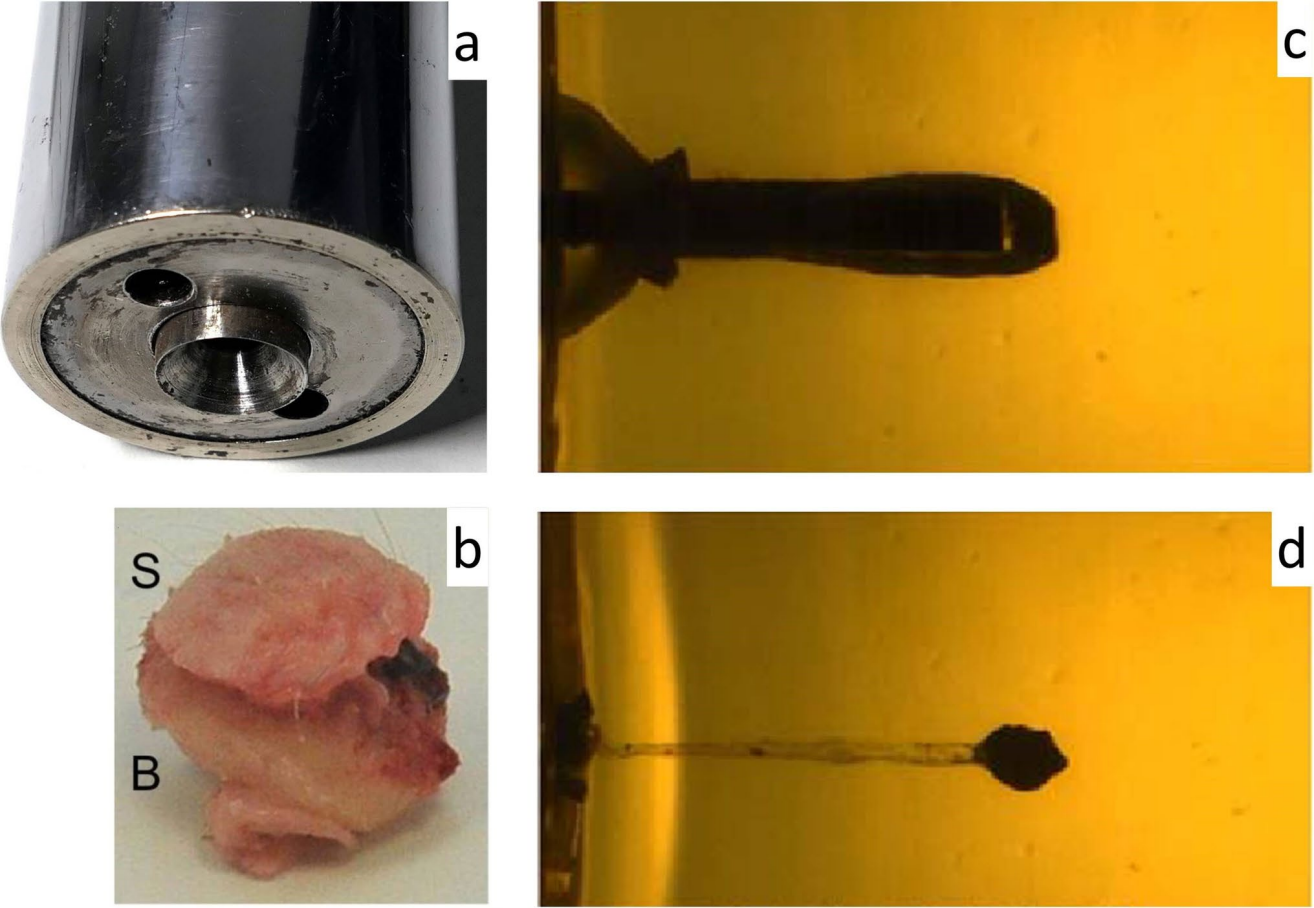
(cf. [123, 126]) Experimental shots with a captive bolt slaughterer's gun. **a** Muzzle plane exhibiting the conically grooved front end of the bolt. **b** Punched-out tissue complex of skin (S) and bone (B). **c** Experimental contact shot with a captive bolt gun to a composite model made of a gelatine block and a synthetic bone plate in front of it. The upper image depicts the bolt at the moment when it reached its maximum penetration depth. The picture below **(d)** shows the position of the punched-out bone fragment after complete retraction of the bolt

Most livestock stunners have bolts with a conically grooved front end. Therefore, they produce sharp-edged skin lesions and circular holes in the underlying flat bone. The punched-out skin-bone complex (‘imprimatum’) is impacted and carried along into the cranial cavity. Test shots to composite models (artificial skin/synthetic bone/gelatine) with accompanying video-documentation confirmed that the imprimatum does not act as a secondary projectile, as its penetration depth is limited by the bolt’s length [126] (Fig. 11).

For a long time, it was erroneously thought that shots from captive bolt guns cannot cause indirect skull fractures away from the actual wound track. Surprisingly, remote fractures were found in some autopsy cases; they were located in especially thin bones such as the orbital plates. Test shots to simulants (glycerin soap, gelatine and skull-brain models) have shown a slight radial displacement even though its extent was not comparable with the TC in shots from conventional guns [125]. So it seems justified to assume that secondary fractures away from the bolt’s path are caused by a hydraulic burst effect within the firm encasement of the skull.

Another area of wound ballistics research is concerned with investigating the effects of less-lethal projectiles (LLPs) as used by law enforcement agencies. The experiments included test shots to different regions of body donors. de Freminville et al. [127] evaluated skin and bone lesions from a grenade launcher firing 40 × 46 mm cartridges. Bir et al. [128] conducted shooting experiments with 12-gauge, fin-stabilized, rubber rocket rounds to measure the energy density required for a 50-% risk of penetration.

Gunshots with Action-1 and -3 ammunition (9 mm Luger) were fired at a part dummy made of textile, pig skin and gelatine [129]. After ricocheting off a concrete floor tile the deformed projectiles penetrated the gelatine block to a depth of between 21 and 37 cm. The ricocheted bullets sprayed a substantial amount of metallic particles onto the textile and further fragments were deposited along the bullet path. The entrance wound morphology varied widely including round lesions. The results proved that ricocheted Action bullets present a serious risk of injury.

Wounds from improvised firearms such as homemade guns may be reconstructed by test shots to models simulating the affected body region, e.g. the victim’s head. To name an example, Tsiatis et al. [130] fired shots at a composite model made up of gelatine and an embedded hemi-sphere consisting of artificial bone. Experimental shots using the same homemade gun as in the case of evaluation produced a wound pattern very similar to the autopsy findings.

For several decades, new approaches to simulation have been adapted. These include, among others, physical/mathematical models (e.g. Sellier’s velocity profile [2], the ‘computer man’ [131] and the ‘rifleman wound model’ [52, 132]) as well as the extensive field of ‘molecular ballistics’ [133]. As these methods do not aim primarily at reconstructing certain wound appearances, we have to leave it at this brief mention.

## Conclusions

- Due to the numerous influencing factors, there is a great variability with regard to the morphological appearance of gunshot injuries. This is one of the main reasons why the medical assessment is prone to error.
- One-component simulants of soft tissue (ballistic gelatine, glycerin soap) or bone (e.g. polyurethane) are well suited for research studies concerning the physical aspects of a bullet’s interaction with a relevant target material (for instance relating to energy absorption and penetration depth).
- Composite models may reproduce more complex conditions such as body regions comprising both soft and hard tissues of various shape and thickness. By firing shots at such models, the wounding effects at the entrance and any exit site as well as along the bullet path can be simulated in a realistic manner.
- To take into account the specific circumstances of an individual case, any modifying factors like intermediate targets can easily be integrated in models used for the reconstruction setup. The same is true for uncommon weapons, peculiar bullet designs, ricochet scenarios and special shooting devices.
